# Antimicrobial Resistance Profiles of *E. coli* Isolated From Pooled Samples of Sick, Farm, and Market Chickens in Nairobi County, Kenya

**DOI:** 10.1155/2024/9921963

**Published:** 2024-10-18

**Authors:** Tino A. Deng, Lilly C. Bebora, Mahacla O. Odongo, Gerald M. Muchemi, Samuel Karuki, Peter K. Gathumi

**Affiliations:** ^1^Department of Veterinary Pathology, Microbiology and Parasitology, University of Nairobi, P.O. Box 29053-00625, Nairobi, Kenya; ^2^Department of Public Health, Pharmacology and Toxicology, University of Nairobi, P.O. Box 29053-00625, Nairobi, Kenya; ^3^Center for Microbiology Research, Kenya Medical Research Institute (KEMRI), P.O Box 43640–00100, Nairobi, Kenya

**Keywords:** antimicrobial resistance, chickens, *E. coli*, Kenya, multidrug resistance

## Abstract

Bacterial antimicrobial resistance (AMR) is a global threat to both human and animal health. This is mainly because the same antimicrobial molecules are used for the treatment and prophylaxis of bacterial diseases in both cases, and about 60% of human pathogens are shared with animals. For effective control of AMR in any country, the current situation has to be established; this is done through surveillance exercises. In Kenya, there is scanty data on the prevailing AMR situation, especially in animals. This paper reports on AMR profiles of 54 *E. coli* strains isolated from chickens in a cross-sectional study, out of which 36/54 (72%) were from clinically ill chickens, 11/54 (22%) were from farm chickens, and 7/54 (9.7%) were from slaughtered chicken, respectively. All 54 isolates exhibited varying antimicrobial resistance profiles with the majority showing resistance to Ampicillin (85.22%), Tetracycline (66.7%), Co-trimoxazole (57.4%), and Streptomycin (40.7%). Very few isolates were resistant to Amoxicillin and Gentamicin (each at 3.7%), Ampicillin (11.1%), and Nalidixic acid (24.1%). A total of 44/54 (81.5%) showed multiple resistance to up to 6 antimicrobial agents. This information will augment current data on the AMR status of bacteria harbored by chickens in Kenya. It will also inform policymakers in their fight against AMR.

## 1. Introduction

Antimicrobials are essential for human and animal health (https://www.reactgroup.org) but need to be used cautiously. Livestock health (including that of poultry and fish) is important for human welfare in two ways: (1) It improves animal welfare, which translates to improved productivity and economic status of stakeholders in the value chain and contributes towards food security. (2) It ensures food safety since it is estimated that about 60% of bacteria that are pathogenic to humans are from animals/animal products [1]. The major problem, concerning the development of antimicrobial resistance, is the fact that some drugs/medicines are used in both humans and animals for treatment or prophylaxis of disease [1–5], and a large percentage of bacteria, both pathogenic or nonpathogenic, are shared between the two groups. Prudent use of antimicrobials in animals is, therefore, important as it will control the transfer of antimicrobial resistance between animals and humans [6]. Indiscriminate usage of antimicrobials, for example, as growth promoters in veterinary medicine [7–10], contributes directly to the emergence and spread of resistance. Indeed, worldwide, it is estimated that 66% of all antimicrobials are used in farm animals and that much of this is intentional routine use (as growth promoters or as prophylactics) to enable farm animals to be kept in poor conditions where disease-causing agents abide [11–14]. In cases of antimicrobial resistance, the resultant food-borne or animal-acquired illness in humans will be less responsive to treatment with respective antimicrobial drug(s).

Since the fight against antimicrobial resistance is of global magnitude [15–17], each country needs to establish and continuously monitor its current status, to harness data for action. In Kenya, as in most developing countries, it is difficult to get a complete picture of the AMR situation, especially in animals because antimicrobial susceptibility testing is not done routinely in diagnostic laboratories (it is only done on specific requests and in specific, although few researches). It is appreciated that several studies on antimicrobial resistance in animals have been carried out in Kenya [18–24]. There are also comprehensive reviews and individual researchers' reports on the situation analysis of AMR in both animals and humans in Kenya [25–27]. This study has determined the extent of antimicrobial resistance in *Escherichia coli* isolated from three groups of chickens. Chickens were used because they are kept and consumed by many Kenyans and there is also a high tendency to use antimicrobials when the chickens are kept under an intensive farming system. *Escherichia coli* was used because it is a common bacterium and also because it is easy to grow and characterize.

## 2. Methods

### 2.1. Study Design, Area, and Sample Chickens

This was a cross-sectional study carried out in Nairobi County, Kenya. It comprised chickens purposively selected from three sources: (1) veterinary poultry clinic at the Department of Veterinary Pathology, Microbiology, and Parasitology, University of Nairobi (a total of 50 sick chickens that were brought to the Poultry clinic for disease diagnosis (postmortem examination; regardless of their disease condition), (2) a poultry commercial farm in Nairobi (50 chickens), and (3) a poultry market/slaughterhouse in Nairobi (72 chickens).

The clinical cases included broilers, layers, and Indigenous chickens from various farms, suffering from various disease conditions (not necessarily caused by *E. coli*) including septicemia, pneumonia, coccidiosis, New castle disease, Gambaro disease, fowl pox, leucosis, nutritional deficiency, aflatoxicosis, yolk-sac infection, helminthiasis, ectoparasites, and trauma (e.g., liver rupture). Like healthy chickens, the clinical cases carry *E. coli* in their guts, as commensals; the isolated *E*. *coli* were, therefore, taken as representatives of strains present in other/healthy chickens in the respective farms. Market birds were mainly of indigenous type and spent layers, brought to the slaughterhouse from various parts of Kenya. The farmed chickens were from a farm that was keeping layers under a slatted floor (wire mesh) system.

### 2.2. Sample Collection, Handling, and Transport

Cloacal swabs were taken from the selected test chickens. They were then placed in Stuart's transport medium (Oxoid Ltd) and transported to the Microbiology laboratory at the department of Veterinary Pathology, Microbiology, and Parasitology, University of Nairobi, for bacterial isolation and identification.

### 2.3. Isolation and Identification of *E. coli*

Isolation of *E. coli* was done by swab-inoculation onto MacConkey agar (Oxoid Ltd), followed by incubation at 37°C overnight. Organisms from lactose-fermenting (pink) colonies were phenotyped and confirmed as *E. coli* through Gram-staining, growth on Eosin Methylene Blue agar, and testing for motility and biochemical reactions, including Indole, Methyl red, Voges Proskauer, Citrate, Urease, and interpretation done using the criteria given in Bergey's Manual of systemic bacteriology [28].

### 2.4. Antimicrobial Susceptibility Testing of the *E. coli* Isolates

Antimicrobial susceptibility testing was done by Agar Disk Diffusion method using Mueller Hinton agar (Oxoid Ltd), as previously described by Bauer et al. [29] and recommended by the Clinical and Laboratory Standards Institute (CLSI) [30]. The *E. coli* isolates were tested for susceptibility against eight antimicrobials that are currently used for treating bacterial infections in both humans and animals in Kenya; including Ampicillin (AMP; 25 *μ*g), Tetracycline (TE; 25 *μ*g), Co-trimoxazole (COT; 25 *μ*g), Streptomycin (S; 10 *μ*g), Nalidixic acid (NA; 30 *μ*g), Amoxicillin (AMC; 30 *μ*g), Gentamicin (GEN; 10 *μ*g), Chloramphenicol (C; 30 *μ*g) (Oxoid, Basingstoke, United Kingdom). After incubation at 37°C overnight, the diameters of the growth-inhibition zones around the discs were measured. *E. coli*, ATCC 25922 [31], was used as the reference strain. Interpretation of the AMR data was done as per CLSI Guidelines [30].

## 3. Results

### 3.1. *Escherichia coli* Isolated From Chickens

*Escherichia coli* organisms were isolated from a total of 54 chickens (31.4%), 36 of them being from the 50 clinical cases (72%); 11 from the 50 farm chickens (22%); and 7 from the 72 market/slaughtered chickens (9.7%).

### 3.2. Antimicrobial Susceptibility/Resistance Test Results for the 54 *E. coli* Isolates

**Figure 1:**
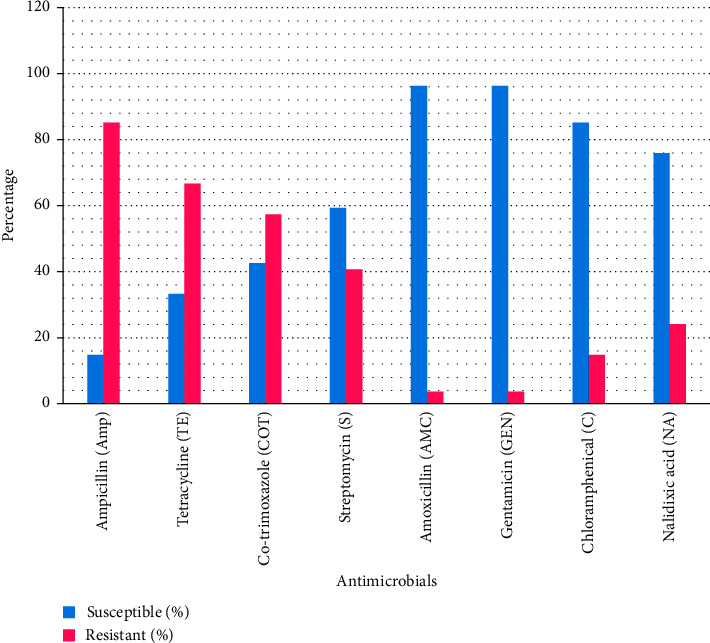
Antimicrobial susceptibility/resistance patterns of *E. coli* isolates (*n* = 54).

Antimicrobial susceptibility test results of the 54 *E. coli* isolates are shown in Table 1.  Figure 1 gives a graphical representation of antimicrobial resistance rates for the test isolates.

The organisms showed the highest resistance to Ampicillin at 85.2%, followed by Tetracycline at 66.7%; Co-trimoxazole at 57.4%; Streptomycin at 40.7%. Low resistances were demonstrated for Nalidixic acid at 24.1% and Chloramphenicol at 14.8% (1 isolate); while high susceptibilities were observed for Amoxicillin and Gentamicin, each at 96.3%. 6 (11.1%) isolates were resistant to one antimicrobial (Ampicillin) only, and 5 (9.3%) were susceptible to all the eight antimicrobials tested, while the rest showed variable resistances ranging from two to six antimicrobials.

### 3.3. Multidrug Resistance in the *E. coli* Isolates

**Figure 2:**
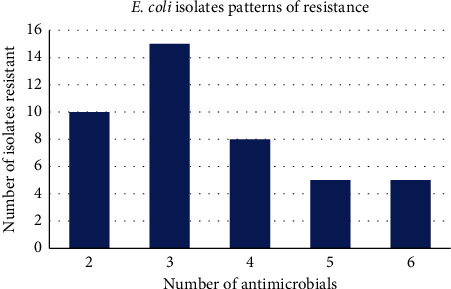
Number of *E. coli* isolates multiresistant to a respective number of antimicrobials (from left to right: 2, 3, 4, 5, and 6 antimicrobials).

Forty-four out of the 54 (81.5%) *E. coli* isolates showed multidrug resistance (resistance to two or more antimicrobials).  Figure 2 presents several organisms resistant to a respective number of antimicrobials; antimicrobial combinations resistant-to are given in Table 1; while Figure 2 shows the number of times an antimicrobial was involved in cases of multidrug resistance among the *E. coli* isolates. Ten (22.7%) of the multidrug-resistant isolates were resistant to two antimicrobials; 15 (34.1%) were resistant to 3 antimicrobials; 8 (18.2%) to 4, while 5 (11.4%) each were resistant to 5 and 6 antimicrobials, respectively (Figure 2). Of the 152 times that the test antimicrobials were included in multidrug combinations, the antimicrobial included most was Ampicillin at 26.3% (40/151); followed by Tetracycline at 23.7% (36/152); Co-trimoxazole at 20.3% (31/152); Streptomycin at 14.5% (22/152); Nalidixic acid at 7.9% (12/152); Chloramphenicol at 5.3% (8/152); Gentamicin at 1.3% (2/152); and lastly Amoxicillin at 0.7% (1/152) (Figure 2).

## 4. Discussion

This study was carried out to determine the current antimicrobial resistance profiles of *E. coli* organisms isolated from chicken cloacae, from selected study sites in Nairobi. There was a low *E. coli* recovery of 31.4% (54/172). This was contrary to what was expected since *E. coli* is one of the most common commensals in the intestinal tracts of both humans and animals; it is also the most commonly isolated bacterium (coprobacterium) from feces [32]. However, this less-than-100%-recovery using the cloacal swab method has been observed in other studies. Ibrahim et al. [33], isolated *E. coli* at 53.4% (269/504); Bebora [34] isolated the organism from 4 lots of chickens at 51.1% (97/133), 46% (98/176), 66% (66/100), and 88% (22/25). This may be due to intermittent shedding of the organisms in feces, as previously documented [35–38]. Shedding is influenced by stress: muscular fatigue, cold, wetness, limitation of food and water, and concurrent infection [36]. Working on *Salmonella typhimurium*, Brownell et al. [36] found that cloacal excretion of the organisms occurred during the first 5 days of infection, after which the excretion dropped considerably. Williams and Whittemore [39] had similar findings; they also concluded that the cloacal swab method was inadequate for the isolation of *Salmonella typhimurium*. The amount of fecal material in the cloacal swab is much less than in the intestinal swab, so there is a higher chance of not picking the organism, even though it is present.

AMR results of this study showed that *E. coli* isolates from the screened chickens were resistant, though at varying levels, to some of the commonly used antimicrobials, predictably because they are cheap and, therefore, affordable to the inhabitants of the study area. The antimicrobial resistance rates were as follows: Ampicillin (85.2%), Tetracycline (66.7%), Co-trimoxazole (57.4%), and Streptomycin (40.7%) (Table 1). The resistance may have developed as a result of high or indiscriminate usage of the antimicrobials in the area; either on the humans or their animals; it may also be as a result of environmental contamination through human/animal movement across the area, through fecal contamination, spitting, or other excrements, or through careless disposal of medicines. This trend of resistance has also been reported in other studies [22, 40–44]. In this study, it was encouraging to find that some *E. coli* strains were still susceptible to the commonly-used antimicrobials; for example, 5 (9.3%) of the isolates were susceptible to all the 8 antimicrobials tested. High susceptibilities were also observed to Amoxicillin and Gentamicin (each at 96.3%), Chloramphenicol (85.2%), and Nalidixic acid (75.9%).

Antimicrobial-resistant bacteria could also have originated from dogs and rats which are normally seen everywhere in human dwellings (especially in informal settlements), in markets, and in farms [45–49]. There is documentation on the presence of zoonotic antimicrobial-resistant bacteria in dogs [44, 50] and rats [22]; hence, these animals can easily and widely disseminate them. Allorechtova et al. [44] specifically looked for ESBL-producing *E. coli* strains in Northern Kenya and demonstrated their presence in humans, dogs, and, to a lesser extent, cats. Comparing genetic profiles of the ESBL-producing *E. coli* isolates, eight isolates from dogs and two isolates from humans gave identical profiles; while a close relationship (> 95%) was found in one human isolate and one cat isolate. This suggests that the spread of resistant bacteria between humans and dogs is a common occurrence; some of these organisms were found to be multidrug resistant. Most farmers practice mixed animal-raising; that is: they keep many types of animals; there is also a close relationship between humans/farmers and their animals; so, resistant bacteria can easily be transferred across the animals and to/from humans.

Many classes of antimicrobials have been used to treat both humans and livestock [4]. They include: *β*-lactams (Penicillins and Cephalosporins); Sulphonamides with or without Trimethoprim; Tetracyclines; Macrolides, Lincosamides, and Streptogramins; and Quinolones including Fluoroquinolones [51]. Classes most used to treat livestock are Penicillin derivatives, such as Ampicillin and Cloxacillin; Sulphonamide, e.g., Tyrosine, used for the treatment of metritis and acute mastitis in cattle, sheep, and goats, enteritis, pneumonia, erysipelas, infectious arthritis in swine, and chronic respiratory disease in chickens [9]. Tetracycline and Co-trimoxazole (containing sulfamethoxazole and trimethoprim) are two most-used antimicrobials for prophylaxis and as growth promoters in livestock rearing, to increase productivity [9]. Most of these are also used in Kenya. Resistance, particularly to the commonly available antimicrobials, poses a major health concern, as alternative therapeutic choices are either unavailable or too expensive to be affordable for most patients.

A high percentage (81.5%; 44/54) of the *E. coli* isolates, in this study, showed multidrug resistance; 10 of them (22.7%) were resistant to two antimicrobials; 15 (34.1%) were resistant to three antimicrobials; 8 (18.2%) to 4, while 5 (11.4%) each were resistant to five and six antimicrobials, respectively (Figure 2). Of the 152 times that the test antimicrobials were included in multidrug combinations, the antimicrobial included most was Ampicillin at 26.3% (40/151); followed by Tetracycline at 23.7% (36/152); Co-trimoxazole at 20.3% (31/152); Streptomycin at 14.5% (22/152); Nalidixic acid at 7.9% (12/152); Chloramphenicol at 5.3% (8/152); Gentamicin at 1.3% (2/152); and lastly Amoxicillin at 0.7% (1/152) (Figure 2). This further demonstrates the resistance pattern as being towards the cheap commonly-used antimicrobials; echoing the worldwide worry towards antimicrobial resistance [2, 15, 16]. Multidrug resistance has been reported by several researchers in Kenya; in animals–Bebora [40], Ombui, Kimotho, and Nduhiu [41], Mapeney et al. [42], Gakuya et al. [22], Kikuvi et al. [43], Allorechtova et al. [44], Igizeneza et al. [52], Wanja et al. [18]; in environment–Wambugu et al. [53], Kutto [21]; in humans–Kariuki et al. [54, 55], Bururia [56], Oundo et al. [57]. It has also been reported by many researchers outside Kenya (Van den Bogaard et al. [58]; Ryu et al. [59]; Adzikey, Huda, and Ali [60]; Nys et al. [61]; Kennedy and Collington [62]; Ulstad et al. [63]; GEN [2].

Increased use of antimicrobials mainly for prophylaxis and as growth promoters in animals in Kenya is encouraged by the increased demand for milk, meat, and eggs, due to increased population and popularization of the products [13, 58]. Most of the antimicrobials are used in intensively kept chickens and pigs, while in other livestock, more antimicrobials are used in the treatment and prevention of mastitis. The Ministry of Agriculture, Livestock, Fisheries, and Irrigation animal census (2017) gives the chicken population to be 48,123,577 (broilers 3,819,515; layers 4,237,188; indigenous 40,067,874). Imprudent use of antimicrobials in chickens in Kenya; coupled with lay administration of the drugs to chickens, facilitated by easy access over the counter [64], is common practice in Kenya (personal observation). Most of the time the unprofessional drug administrators (farmers, etc.) are deprived of instructions and, hence tend to do so incorrectly, or purchase the wrong drug [65]. The situation is made worse by human doctors and veterinarians who tend to use antimicrobials as a cover for any secondary bacterial infection; they use the assurance that: “if it is broad-spectrum, it can shoot better” [66]. This is coupled with the increased use of antimicrobials in humans, mainly to treat respiratory, enteric, and hospital-acquired infections [56, 57, 67, 68]. There is also increased usage of antimicrobials, especially Tetracycline and Co-trimoxazole, in HIV-AIDS patients, to treat infections related to Acquired Immunodeficiency Syndrome (AIDS) in humans [8]. The number of people living with HIV/AIDS in Kenya is estimated to be 1.6 million (UNAIDS, 2017 Data Book; National Aids Control Council report 2018). The wide, sometimes unjustified use of antimicrobials in humans and animals in Kenya may explain the high occurrence of antimicrobial resistance in the *E. coli* strains tested in this study.

## 5. Conclusion

This study has demonstrated the carriage of antimicrobial-resistant *E. coli* in Kenyan chicken; most of them showing multidrug resistances ranging from two to six antimicrobials; the number could have been even higher if more antimicrobials were tested. Data from this study is expected to augment the AMR baseline data already collected for Kenya, it will also inform policymakers in their fight against AMR.

## Figures and Tables

**Table 1:** Multidrug resistant patterns demonstrated by the test isolates.

**Number of antimicrobials**	**Number of isolates resistant**	**Type of antimicrobials**
2	1	COT-TE
	1	S-TE
	1	COT-AMP
	1	S-AMP
	6	TE-AMP

3	1	NA-S-TE
	1	C-TE-AMP
	1	C-S-AMP
	4	COT-S-AMP
	8	COT-TE-AMP

4	1	NA-S-TE-AMP
	2	COT-NA-TE-AMP
	5	COT-S-TE-AMP

5	1	COT-NA-GEN-TE-AMP
	2	COT-NA-S-TE-AMP
	2	COT-C-S-TE-AMP

6	1	COT-C-NA-GEN-TE-AMP
	1	COT-NA-S-AMC-TE-AMP
	3	COT-C-NA-S-TE-AMP

Abbreviations: AMC, Amoxicillin; AMP, Ampicillin; COT, Co-trimoxazole; C, Chloramphenicol; GEN, Gentamicin; NA, Nalidixic acid; S, Streptomycin; TE, Tetracycline.

## Data Availability

The data used and analysed in this study are obtainable from the corresponding author on rational demand.
